# Potenzieller Einfluss der Reizschwellenhöhe des nozizeptiven Flexorenreflex (NFRT) auf die Mortalitäts- und Delirinzidenz beim kritisch kranken Patienten: eine retrospektive Kohortenanalyse

**DOI:** 10.1007/s00101-022-01206-8

**Published:** 2022-09-27

**Authors:** B. Schick, S. Schmid, B. Mayer, D. Wagner, S. Walter, S. Gruss, B. Jungwirth, E. Barth

**Affiliations:** 1 Klinik für Anästhesiologie und Intensivmedizin, Sektion Interdisziplinäre Operative Intensivmedizin, Universitätsklinikum Ulm, Albert-Einstein-Allee 23, 89081 Ulm, Deutschland; 2 Institut für Epidemiologie und Medizinische Biometrie, Universität Ulm, Schwabstr. 13, 89075 Ulm, Deutschland; 3 Klinik für Psychosomatische Medizin und Psychotherapie, Sektion medizinische Psychologie, Universität Ulm, Frauensteige 6, 89075 Ulm, Deutschland

**Keywords:** Analgesie und Sedierung, Intensivpatient, Behavioral Pain Skala, Delirium, Schmerz, Analgesia and sedation, Intensive care patient, Behavioral Pain Scale, Delirium, Pain

## Abstract

**Hintergrund:**

Mortalität und Delirinzidenz werden beim kritisch kranken Patienten durch das Analgosedierungsregime beeinflusst. Je tiefer die Sedierung, je höher die Dosis applizierter Analgetika, desto schwieriger ist die Einschätzung von Schmerz und Sedierungsgrad. Daher gewinnen apparative Messverfahren, wie die Messung der Reizschwelle des nozizeptiven Flexorenreflexes (NFRT), zunehmend an Bedeutung.

**Ziel der Arbeit:**

Ziel der vorliegenden Studie ist es, eine mögliche Assoziation zwischen der Höhe des nozizeptiven Flexorenreflexes, der Mortalität und dem Auftreten eines Delirs zu untersuchen.

**Material und Methodik:**

Durch die retrospektive Analyse eines 57 Intensivpatienten umfassenden Pilotdatensatzes der interdisziplinären operativen Intensivstation des Universitätsklinikums Ulm, erhoben zwischen November 2018 und März 2020, wurde in einem adjustierten logistischen Regressionsmodell eine mögliche Assoziation zwischen NFRT, Mortalität und Delirinzidenz berechnet. Je nach Cut-off-Wert ergeben sich Reizschwellenkorridore mit folgenden Vergleichspaaren: < 20 mA vs. 20–40 mA/20–50 mA/20–60 mA, > 40 mA vs. 20–40 mA, > 50 mA vs. 20–50 mA, > 60 mA vs. 20–60 mA. Die Ergebnisdarstellung erfolgt als Odds Ratios, bereinigt um Alter, Geschlecht, Größe, TISS-28, SAPS II, RASS, BPS und die verwendeten Analgetika. Die Schmerzerfassung erfolgte in der untersuchten Gruppe standardisiert mittels der Behavioral Pain Scale sowie ergänzend durch die NFRT-Messung.

**Ergebnisse:**

Es konnte eine statistisch nicht signifikante Tendenz zu einer Mortalitätszunahme bei einer NFRT > 50 mA gegenüber dem Reizschwellenkorridor von 20–50 mA ermittelt werden (OR 3.3, KI: 0,89–12.43, *p* = 0,07). Eine Tendenz zu einer Reduktion der Delirhäufigkeit trat bei einer NFRT < 20 mA gegenüber einem Reizschwellenkorridor von 20–40 mA auf (OR 0.40, KI: 0,18–0,92, *p* = 0,03).

**Diskussion:**

Anhand der Höhe der NFRT kann zum aktuellen Zeitpunkt keine Empfehlung zur Anpassung des verwendeten Analgosedierungsregimes beim kritisch kranken, nichtmitteilungsfähigen Intensivpatienten gegeben werden. Die Beobachtung einer Tendenz hin zu einer Zunahme der Mortalität bei hohen Reizschwellen bzw. einer Reduktion des Auftretens eines Delirs bei niedrigen Reizschwellen muss in standardisierten Studien überprüft werden.

**Zusatzmaterial online:**

Die Online-Version dieses Beitrags (10.1007/s00101-022-01206-8) enthält weitere Tabellen.

## Abstract (en)

**Background:**

Mortality and delirium in critically ill patients are affected by the provided analgesics and sedatives. The deeper the sedation and the higher the dose of analgesics applied, the more difficult it is to assess pain and the depth of sedation. Therefore, instrumental measurement methods, such as the measurement of the stimulus threshold of the nociceptive flexion reflex (NFRT), are becoming increasingly more important.

**Objective:**

The aim of the present study is to investigate a potential association between the level of the nociceptive flexion reflex, mortality, and the occurrence of delirium.

**Material and methods:**

By retrospectively analyzing a pilot data set of 57 ICU patients from the interdisciplinary surgical ICU of Ulm University Hospital surveyed between 11/2018 and 03/2020, a possible association between the NFRT, mortality, and the occurrence of delirium was calculated in an adjusted logistic regression model. Depending on the cut-off value, the stimulus threshold corridors result in the following comparison pairs: < 20 mA vs. 20–40 mA/20–50 mA/20–60 mA, > 40 mA vs. 20–40 mA, > 50 mA vs. 20–50 mA and > 60 mA vs. 20–60 mA. Results are presented as odds ratios (OR) adjusted for age, sex, height, TISS-28, SAPS II, RASS, BPS, and applied analgesics. Pain assessment was performed, in addition to the Behavioral Pain scale, ≥ 3 times daily by measuring NFRT.

**Results:**

A statistically nonsignificant tendency for an increase in mortality incidence occurred with an NFRT > 50 mA, versus a stimulus threshold corridor of 20–50 mA (OR 3.3, CI: 0.89–12.43, *p* = 0.07). A trend toward a reduction in delirium incidence occurred at an NFRT < 20 mA, versus a stimulus threshold corridor of 20–40 mA (OR 0.40, CI: 0.18–0.92, *p* = 0.03).

**Conclusion:**

Based on the level of the NFRT, no recommendation can be made at this point to adjust the analgesic regimen of critically ill patients, who are unable to communicate. The observation of a tendency towards an increase in mortality at high stimulus thresholds or a reduction in the occurrence of delirium at low stimulus thresholds of the NFRT must be verified in standardized studies.

## Hintergrund

Knapp über 10 % der kritisch kranken Patienten versterben innerhalb eines Jahres nach einer intensivstationären Therapie [4]. Besonders betroffen sind Patienten, die ein Delir erleiden [21]. Sowohl die Mortalität als auch das Auftreten eines Delirs werden dabei durch Art und Dosierung der applizierten Analgetika und Hypnotika im Rahmen der intensivstationären Komplexbehandlung beeinflusst [2, 26]. Besonderes Augenmerk muss auf die Gruppe der Intensivpatienten gelegt werden, die ihre Schmerzen nicht selbst kommunizieren können und eine tiefe Analgosedierung benötigen. Zur Schmerzeinschätzung mittels etablierter Schmerzerfassungsskalen kommt bei dieser Patientengruppe die Notwendigkeit, Parameter wie Herzfrequenz, Tränenfluss etc., die durch verschiedene Faktoren beeinflusst werden können, in die Interpretation der Schmerzen einzubeziehen [1, 3, 19]. Dies birgt das Risiko einer Fehleinschätzung des Analgesieniveaus. Daher gewinnen Verfahren zur Objektivierung nozizeptiver Vorgänge zunehmend an Bedeutung [8, 9, 11, 18, 34]. Eines dieser Verfahren ist die Messung der RIII-Komponente der Reizschwelle des nozizeptiven Flexorenreflexes (NFRT). Die Höhe der Reizschwelle korreliert dabei u. a. mit der Wahrscheinlichkeit einer Reaktion auf einen schmerzhaften Stimulus [8, 9]. Hohe Reizschwellen sind bei hohen Analgetikadosierungen zu erwarten, niedrige Reizschwellen entsprechend bei einer geringeren Analgetikadosis. Weiterhin besteht eine Assoziation zwischen Sedierungstiefe, Schmerzniveau und NFRT [8, 9, 11, 15, 23, 31, 34]. Vor allem bei tiefer Analgosedierung ist nicht nur die Schmerzeinschätzung limitiert, sondern auch ein strukturiertes Delirscreening nicht immer zuverlässig möglich [35]. Daher war das Ziel der vorliegenden Studie zu überprüfen, ob eine Assoziation zwischen der Höhe der NFRT als Surrogatparameter nozizeptiver Vorgänge und indirekt des Analgosedierungsniveaus und der Mortalität in einem heterogenen Kollektiv kritisch kranker, analgosedierter, mechanisch beatmeter und nichtmitteilungsfähiger Patienten besteht. Weiterhin wurde die Hypothese überprüft, ob die Höhe der NFRT mit dem Auftreten eines Delirs korreliert.

## Methodik

### Studiendesign

Nach positivem Votum der Ethikkommission der Universität Ulm (Amendment 2 zum Ethikantrag 284/18) erfolgte eine retrospektive Kohortenanalyse eines Pilotdatensatzes einer zwischen November 2018 und März 2020 am Universitätsklinikum Ulm, Klinik für Anästhesiologie und Intensivmedizin (Interdisziplinäre Operative Intensivstation), durchgeführten prospektiven Studie. Dabei wurden 144 kritisch kranke, analgosedierte, mechanisch beatmete und nichtmitteilungsfähige Patienten eingeschlossen. Alle Patienten wurden auf der interdisziplinären operativen Intensivstation behandelt, was die Heterogenität des Datensatzes erklärt. Die Patienten wurden mittels einfacher Randomisierung in 2 Gruppen eingeteilt. In Gruppe A (Grundlage der retrospektiven Datenanalyse) wurde ergänzend zur Schmerzerfassung mittels der Behavioral Pain Scale (BPS) die NFRT gemessen. Gruppe B (Daten nicht berichtet) erhielt eine Schmerzerfassung ausschließlich mittels der BPS.

## Messung der NFRT

Die Messung der Reizschwelle des NFR zielt darauf ab, die Erregbarkeit der spinalen Anteile des Schmerzverarbeitungssystems zu untersuchen. Der afferente Reflexbogen besteht aus A_δ_- und C‑Fasern. Die elektrische Stimulation des für die Auslösung des Reflexes verantwortlichen N. tibialis erfolgt über an der medialen Plantarfläche angebrachte Elektroden. Die stimulierten A_δ_- und C‑Fasern vermitteln die Reizweiterleitung an das Rückenmark. Dort erfolgt die polysynaptische Verschaltung des Reizes, was zu einer Aktivierung von Motoneuronen (efferenter Reflexanteil) führt. Die resultierende Reizantwort wird über ein Oberflächenelektromyogramm am M. tibialis anterior erfasst [10, 14, 15]. Für eine umfassende Erklärung wird an dieser Stelle auf die zitierten Referenzen verwiesen. Aufgrund der inkonsistenten Datenlage wurden folgende Parameter hinsichtlich ihres Einflusses auf die NFRT in die Regressionsanalyse einbezogen (Tab. 1):

**Table 1:** 

Alter	Mit zunehmendem Alter Anstieg der NFRT [22]
Geschlecht	Möglicherweise geringere NFRT bei Frauen [13]
Analgetika	Dosisabhängiger Anstieg der NFRT [8–10]
SAPS II, TISS 28	Erkrankungsschwere assoziiert mit Critical-Illness-Polyneuro- und Myopathie – potenzieller Einfluss auf die NFRT durch Störung der Nervenleitgeschwindigkeit [6, 16, 30]
Glucosekonzentration Insulindosis	Hyperglykämie als Risikofaktor für die Entstehung einer Critical-Illness-Polyneuro- und Myopathie [25, 29]
RASS	Indirekte Assoziation zwischen Sedierungstiefe und Höhe der NFRT [23]
BPS	Negative Assoziation zwischen BPS und NFRT [23]

*SAPS II* Simplified Acute Physiology Score II, *TISS-28* Therapeutic Intervention Scoring System, *RASS* Richmond Agitation Sedation Scale, *BPS* Behavioral Pain Scale

Die erste Messung, sowohl der NFRT als auch der BPS, erfolgte innerhalb von 12 h nach Aufnahme auf die Intensivstation. Die Messungen wurden bis zur Extubation und zur Beendigung der Analgosedierung der Patienten in individuellen Abständen durchgeführt. Wesentliches Kriterium für die Messungen war ein mindestens 30-minütiger Abstand zu vorausgegangenen medizinischen/pflegerischen Maßnahmen. Dies sollte eine mögliche Beeinflussung durch potenziell schmerzhafte Stimuli und damit eine Veränderung der NFRT weitestgehend reduzieren, um eine Vergleichbarkeit der Daten zu gewährleisten.

### Analgosedierungsregime

#### Analgetika

Die Patienten erhielten Remi- oder Sufentanil in einer körpergewichtsadaptierten Dosierung, mit dem Ziel, einen BPS-Wert von 3 bis 4 (keine bis leichte Schmerzen) zu erreichen. Ergänzend erhielten die Patienten, sofern keine Kontraindikationen bestanden, Metamizol über eine Spritzenpumpe in einer Dosierung von 4 g/24 h.

#### Hypnotika

Als sedierende Medikation erhielten alle Patienten entweder Propofol oder Lormetazepam körpergewichtsadaptiert infundiert. Ziel war, eine Sedierungstiefe mit einem RASS-Wert ≥ −3 zu erreichen. Eine tiefere Sedierung wurde bei entsprechender medizinischer Indikation, wie beispielsweise einem schweren Schädel-Hirn-Trauma oder einem ARDS, angestrebt. Wir verweisen an dieser Stelle auf die bereits veröffentlichen Daten zur Abhängigkeit der NFRT vom gewählten Analgosedierungsregime [23].

Die Ziele der retrospektiven Analyse wurden wie folgt definiert:

- Besteht zwischen der Höhe der NFRT, gemessen innerhalb der ersten 4 Tage nach Aufnahme auf die Intensivstation und der Mortalität, definiert als „verstorben während der intensivmedizinischen Behandlung“, eine Assoziation?
- Besteht zwischen der Höhe der NFRT, gemessen innerhalb der ersten 4 Tage nach Aufnahme auf die Intensivstation und der Delirinzidenz während des intensivstationären Aufenthaltes eine Assoziation?

Die Delirdiagnose (hypo-, hyperaktiv, Mischtyp) wurde aus der intensivstationären Verlaufsdokumentation extrahiert. Der dazu durchgeführte CAM-ICU wurde 3‑mal täglich oder bei einer klinischen Veränderung durch geschultes ärztliches oder pflegerisches Personal erhoben. Der Endpunkt Mortalität wurde definiert als „verstorben während des intensivstationären Aufenthaltes“ und ebenfalls der Patientendokumentation entnommen.

Die Studie wurde in Übereinstimmung mit der Deklaration von Helsinki durchgeführt.

### Reizschwellenmodulation

Bislang existieren kaum valide Daten, die anhand der Höhe der NFRT eine Bewertung der Analgesiequalität erlauben. Die verfügbare Evidenz zeigt, dass bei einer NFRT < 20 mA eine geringe Analgesie angenommen werden kann [11, 15]. Daher wurde im Rahmen der vorliegenden Studie ein Wert von 20 mA als untere Grenze für eine analgetisch ausreichende Behandlung angesehen. Ein oberer Grenzwert der NFRT, ab dem von einer zu exzessiven Analgesie gesprochen werden kann, konnte bislang nicht definiert werden. Für die aktuelle Studie wurden daher 3 verschiedene obere Reizschwellen (> 40 mA, > 50 mA, > 60 mA) untersucht. Entsprechend den gewählten Cut-off-Werten ergeben sich Reizschwellenkorridore mit folgenden Vergleichspaaren:

- < 20 mA vs. 20–40 mA/20–50 mA/20–60 mA,
- > 40 mA vs. 20–40 mA,
- > 50 mA vs. 20–50 mA,
- > 60 mA vs. 20–60 mA.

Für die Endpunkte „Mortalität“ und „Delir“ gibt es bislang keine definierten Schwellenwerte der NFRT.

## Fallzahl und Poweranalyse

Die Fallzahlberechnung der prospektiven Studie, als Grundlage der vorliegenden retrospektiven Analyse, erfolgte mittels GPower 3.1. Je Gruppe wurden 105 Patienten (NFRT + BPS/BPS solo) berechnet, um eine statistische Power von 80 % mit einen zweiseitigen α‑Niveau < 0,05 und einer Effektstärke von d = 0,5 zu erreichen. Aufgrund der SARS-CoV-2-bedingten Einschränkungen musste die Studie vor Erreichen der endgültigen Fallzahl beendet werden.

### Statistische Analyse

Die Datenerfassung erfolgte mit Microsoft EXCEL 2019® (Microsoft Corp., Redmond, WA, USA). Die statistische Auswertung wurde mit SAS®, Version 9.4 (SAS Institute GmbH, Heidelberg, Deutschland) durchgeführt. Für die deskriptive Analyse kategorialer Merkmale wurden Häufigkeiten berechnet, alle metrisch-skalierten Merkmale wurden mittels Median und Interquartilsabstand analysiert. Die Analyse der verbundenen Stichproben erfolgte nach Testung auf Normalverteilung mittels Shapiro-Wilk-Test entweder mit dem gepaarten t‑Test bzw. mit dem Wilcoxon-Rangsummentest für verbundene Stichproben. Der Datensatz wurde dahingehend analysiert, dass jeweils Wertepaare für die Tage 1 bis 3 verglichen wurden. Patienten, bei denen Messwerte nur bis Tag 2 generiert wurden, konnten somit nicht in die Auswertung eingeschlossen werden. Der Messtag 4 wurde aufgrund zu geringer Messwerte aus der Analyse der verbundenen Stichprobe ausgeschlossen. Die unverbundenen Stichproben wurden nach Testung auf Normalverteilung entweder mittels t‑Test für unabhängige Stichproben oder Mann-Whitney-U-Test analysiert. Für die Evaluierung der Assoziation der NFRT mit Mortalität und Auftreten eines Delirs wurde aufgrund wiederholter Messungen pro Patient ein Modell generalisierter Schätzgleichungen (GEE) verwendet. Auf diese Weise konnten die jeweils dichotomen Endpunkte (Mortalität und Auftreten eines Delirs) im Sinne einer logistischen Regression analysiert werden, mit der zusätzlichen Möglichkeit einer Adjustierung für demografische und klinische Parameter der Patienten. Die Interpretation der Ergebnisse erfolgt dementsprechend über die Odds Ratio (OR) mit zugehörigem 95 %-Konfidenzintervall (KI). Für alle Analysen wurde ein exploratives, zweiseitiges Typ-1-Fehlerniveau von 5 % angenommen.

## Ergebnisse

**Figure 1:**
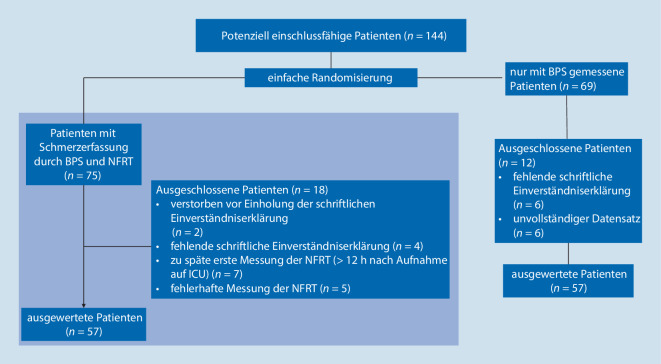

Während des Untersuchungszeitraums wurde bei 75 Patienten ergänzend zur BPS die NFRT erfasst. Davon konnten 57 Patienten in die Datenauswertung eingeschlossen werden (Abb. 1). Die demografischen Angaben sind in Tab. 2 abgebildet. Die Anzahl an Messungen der NFRT war zwischen den Patienten unterschiedlich häufig (Zusatzmaterial online: Tab. 1).

**Table 2:** 

Variable	Gesamtkollektiv
**Anzahl an Patienten**	*n* = 57
**Alter (Jahre**^**a**^**)**	61,0 (54,0–72,0)
**Geschlecht, *n* (%)**	
Männlich	44 (77,2)
Weiblich	13 (22,8)
**Aufenthaltsdauer auf der Intensivstation (Tage**^**a**^**)**	10,0 (5,0–19,0)
**Intensivmedizinische Komplexbehandlung**	
SAPS II^a^	36,0 (29,0–45,0)
TISS-28^a^	19,0 (14,0–24,0)
Remifentanil mg/d Median^a^	4,8 (0,0–7,2)
Sufentanil mg/Tag, Median^a^	0,3 (0,1–0,5)
Propofol g/Tag, Median^a^	4,8 (3,4–4,8)
**Mortalität, *n* (%)**	6 (10,5)
**Delirium, *n* (%)**	17 (29,8)
*Hyperaktiv*	12 (21,1)
*Hypoaktiv*	1 (1,8)
*Mischtyp*	4 (7,0)
**Aufnahmegrund auf die Intensivstation, *n* (%)**	
Neurochirurgie	13 (22,8)
Viszeralchirurgie	15 (26,3)
Trauma	5 (8,8)
Kardiochirurgie	2 (3,5)
Gefäßchirurgie	4 (7,0)
Thoraxchirurgie	5 (8,8)
Akutes Lungenversagen	5 (8,8)
Urologie	7 (12,3)
Mund‑, Kiefer‑, Gesichtschirurgie	1 (1, 8)

*SAPS II* Simplified Acute Physiology Score, *TISS-28* Therapeutic Intervention Scoring System-28

^a^Median und Interquartilsabstand

### Assoziation zwischen NFRT und Mortalität

**Figure 2:**
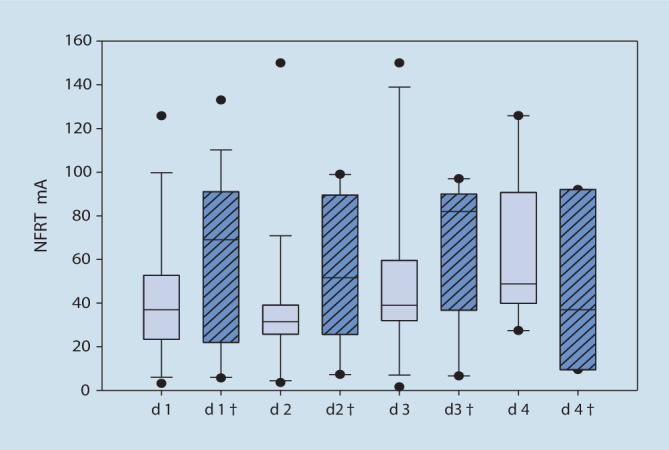

In der Gruppe der verstorbenen Patienten war die gemessene Höhe der NFRT-Werte zwischen den Tagen 1 bis 3 vergleichbar hoch (d_1_/_2_: t = 1,19, *p* = 0,274, *n* = 8, d_2/3_: Mdn d_2_ = 69,30, Mdn d_3_ = 82,00, z = 0,340, *p* = 0,813, *n* = 8, d_1/3_: t = 0,81, *p* = 0,442, *n* = 8). Bei den nichtverstorbenen Patienten zeigten sich zwischen den Messtagen 1 bis 3 ebenfalls keine statistisch signifikanten Unterschiede der NFRTs (d_1_/_2_: t = −0,021, *p* = 0,984, *n* = 30, d_2/3_: Mdn d_2_ = 31,50, Mdn d_3_ = 37,50, z = 1,60, *p* = 0,113, d_1/3_: t = −1,42, *p* = 0,167, *n* = 30; Abb. 2). Auch im direkten Vergleich der beiden Gruppen konnte zwischen den Messtagen kein statistisch signifikanter Unterschied der NFRT festgestellt werden (Zusatzmaterial online: Tab. 2).

**Figure 3:**
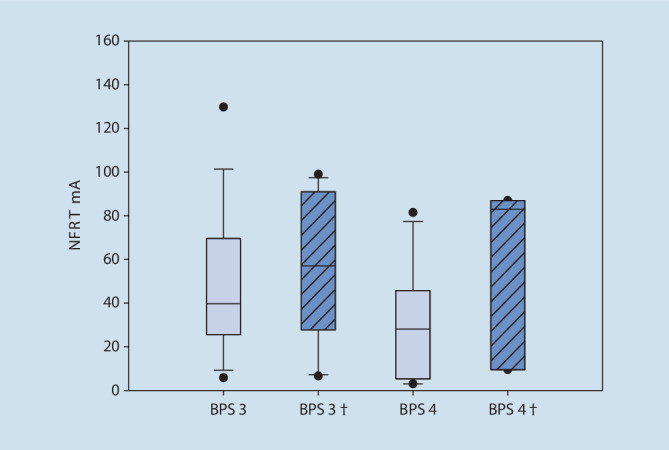

In Abb. 3 wurden die bei einer BPS von 3 bzw. 4 gemessenen NFRT-Werte der nichtverstorbenen und verstorbenen Patienten gegenübergestellt. Es zeigt sich sowohl zwischen den Gruppen als auch innerhalb der Gruppen kein statistisch signifikanter Unterschied in der Höhe der NFRT (nichtverstorben – NFRT (mA) BPS_3/4_:t = 1,81, *p* = 0,091, *n* = 16, Verstorben – NFRT(mA) BPS_3/4_:t = 1,14,*p* = 0,37, *n* = 3). Im direkten Vergleich der beiden Gruppen zeigt sich jedoch, dass die verstorbenen Patienten bei einer BPS von 3 bzw. 4 statistisch nicht signifikante, aber höhere NFRT-Werte als nichtverstorbene Patienten hatten (NFRT (mA), 95 % KI bei BPS 3: nichtverstorben: 39,7 [25,6–69,5] vs. verstorben: 57,0 [27,8–91,0], *p* = 0,367, BPS 4: nichtverstorben: 28,1 [5,25–45,73] vs. verstorben: 83,0 [9,5–87,0], *p* = 0,105). Wie in Tab. 3 dargestellt, erhielt die Gruppe der verstorbenen Patienten statistisch signifikant mehr Analgetika sowie Lormetazepam und war konsekutiv tiefer sediert. Weiterhin waren die verstorbenen Patienten statistisch signifikant schwerer erkrankt und hatten höhere Blutglucosekonzentrationen bzw. einen statistisch signifikant höheren Insulinbedarf.

**Table 3:** 

	Nichtverstorben *n* = 47	Verstorben *n* = 6	*p*-Wert
*Analgesie*			
Sufentanil (µg/kgKG und h)	0,14 [0,11–0,18]	0,20 [0,2–0,20]	0,006^b^
Remifentanil (µg/kgKG und min)	0,06 [0,04–0,08]	0,03 [0,03–0,06]	< 0,001^a^
Metamizol (g/Tag)	4,0 [4,0–4,0]	4,0 [4,0–4,0]	0,640
BPS, Median, Min – Max	3,0 [3,0–9,0]	3,0 [3,0–4,0]	0,177^a^
*Sedierung*			
Propofol (mg/kgKG und h)	2,22 [1,78–2,86]	1,77 [1,13–3,00]	0,130^a^
Lormetazepam (mg/kgKG und h)	0,007 [0,003–0,008]	0,008 [0,008–0,009]	< 0,001^a^
RASS	−3,0 [−4,0 bis −2,0]	−4,0 [−5,0 bis −3,0]	< 0,001^a^
*Erkrankungsschwere*			
TISS 28	15,0 [10–20]	23,0 [18,0–29,0]	0,002^a^
SAPS II	37,0 [29,0–41,0]	40,0 [28,50–50,0]	0,042^b^
*BZ und Insulinbedarf*			
Glucose (mg/dl)	133,0 [117,0–152,3]	145,0 [130,0–164,0]	0,002^a^
Insulin (IE/h)	1,75 [0,5–4,0]	5,0 [2,0–6,0]	< 0,001^a^

*BPS* Behavioral Pains Scale, *TISS 28* Therapeutic Intervention Scoring System, *SAPS II* Simplified Acute Physiology Score II

^a^Mann-Whitney-U-Test

^b^t‑Test. Ergebnisse sind als Median und 95 %-Konfidenzintervall angegeben

## Ergebnisse der logistischen Regression – Assoziation zwischen NFRT und Mortalität

Wurde eine NFRT > 50 mA gegenüber dem Reizschwellenkorridor von 20–50 mA gemessen, so konnte im Regressionsmodell eine Tendenz zu einer nichtsignifikanten Zunahme der Mortalität um den Faktor 3,3 ermittelt werden (OR = 3,32, 95 %-KI 0,88–12,43, *p* = 0,077). Dieser Effekt blieb nach Adjustierung für verschiedene potenzielle Störvariablen bestehen (Zusatzmaterial online: Tab. 3). Bei einer NFRT > 60 mA, verglichen mit dem Reizschwellenkorridor von 20–60 mA, kam es zu einer nichtsignifikanten Zunahme der Mortalität um den Faktor 4,1 (adjustiert nach SAPS II, OR: 4,1, KI: 0,97–17,72, *p* = 0,055, Zusatzmaterial online: Tab. 3). Bei einer geringeren NFRT von > 40 mA, verglichen mit einem Reizschwellenkorridor von 20–40 mA, war die Veränderung der Mortalität im Sinne einer Zunahme, nach Adjustierung für die potenziellen Störgrößen, geringer (Zusatzmaterial online: Tab. 3). Bei einer NFRT < 20 mA zeigte sich, verglichen mit den Reizschwellenkorridoren 20–40 mA, 20–50 mA und 20–60 mA, keine Zunahme der Mortalität (Zusatzmaterial online: Tab. 3 ).

Die Delirinzidenz im Rahmen der intensivmedizinischen Komplexbehandlung ist u. a. vom gewählten Analgosedierungsregime abhängig. Daher wurde ihre Assoziation mit der Höhe der NFRT in der vorliegenden Studie untersucht.

### Assoziation zwischen der Höhe der NFRT und dem Auftreten eines Delirs

**Figure 4:**
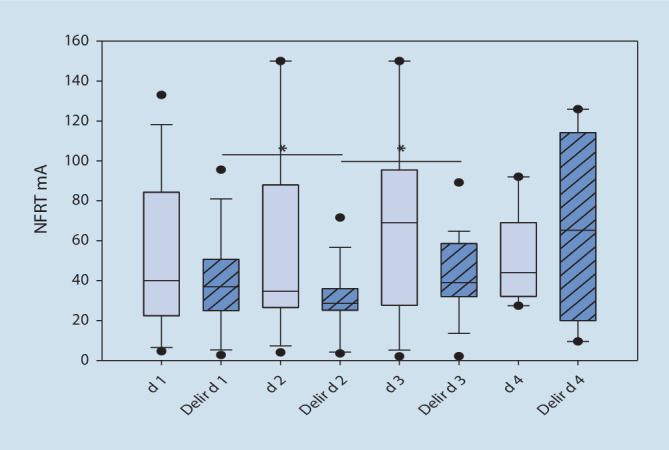

**Figure 5:**
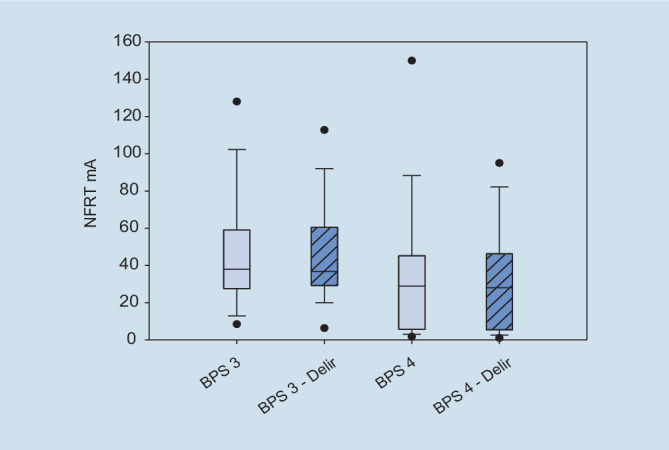

Innerhalb der Gruppe der deliranten Patienten konnte zwischen den Messtagen 1 und 2 sowie 2 und 3 ein statistisch signifikanter Unterschied in der Höhe der NFRT nachgewiesen werden (Abb. 4, d_1_/_2_: Mdn d_1_ = 36,90, Mdn d_2_ = 27,30, z = −3,05, *p* = 0,001, d_2/3_: t = −3,38, *p* = 0,004, *n* = 17, d_1_/_3_: t = 0,23, *p* = 0,820, *n* = 17). Bei den nichtdeliranten Patienten kam es zwischen den Messtagen zu keinen statistisch signifikanten Unterschieden (d_1_/_2_: t = −0,71, *p* = 0,488, *n* = 16, d_2_/_3_: t = −0,81, *p* = 0,429, *n* = 16, d_1_/_3_: t = −1,34, *p* = 0,199, *n* = 16). Im direkten Vergleich beider Gruppen ergaben sich ebenfalls keine Unterschiede in der Höhe der NFRT (Zusatzmaterial online: Tab. 2). In Abb. 5 wurden die bei einer BPS von 3 bzw. 4 gemessenen NFRT-Werte der nichtdeliranten und deliranten Patienten gegenübergestellt. Innerhalb der Gruppe der nichtdeliranten sowie der deliranten Patienten konnte zwischen den gemessenen BPS-/NFRT-Werten kein statistisch signifikanter Unterschied nachgewiesen werden (Nichtdelir: NFRT (mA) BPS_3/4_: Mdn_BPS3_: 40,10, Mdn_BPS4_: 28,90, z = −2,13, *p* = 0,034, Delir: NFRT (mA) BPS_3/4_: Mdn_BPS3_: 40,80, Mdn_BPS4_: 28,00, z = −1,40, *p* = 0,174). Ein direkter Vergleich der Gruppen erbrachte ebenfalls keine statistisch signifikanten Unterschiede in den gemessenen NFRT-BPS-Werten (NFRT (mA), 95 %-KI bei BPS 3: Nichtdelir: 37,97 [27,62–59,05] vs. Delir: 40,10 [31,15–55,30], *p* = 0,773, BPS 4: Nichtdelir: 28,9 [5,75–45,17] vs. Delir: 28,0 [5,75–46,30], *p* = 0,857). Delirante Patienten erhielten, wie in Tab. 4 ersichtlich, statistisch höhere Dosierung an Remifentanil. Nichtdeliranten Patienten wurde statistisch signifikant mehr Lormetazepam appliziert, ohne messbaren Unterschied der Sedierungstiefe zwischen den Vergleichsgruppen.

**Table 4:** 

	Nichtdelir (*n* = 35)	Delir (*n* = 17)	*p*-Wert
*Analgesie*			
Sufentanil (µg/kgKG und h)	0,13 [0,09–0,18]	0,11 [0,11–0,12]	0,956^b^
Remifentanil (µg/kgKG und min)	0,04 [0,03–0,06]	0,08 [0,05–0,08]	< 0,001^a^
Metamizol (g/Tag)	4,0 [4,0–4,0]	4,0 [4,0–4,0]	0,460
BPS, Median. Min – Max	3,0 [3,0–5,0]	3,0 [3,0–9,0]	0,433^a^
*Sedierung*			
Propofol (mg/kgKG und h)	2,2 [1,66–2,86]	2,0 [1,56–2,50]	0,196^a^
Lormetazepam (mg/kgKG und h)	0,008 [0,005–0,008]	0,003 [0,003–0,003]	< 0,001^a^
RASS	−4,0 [−5,0–−3,0]	−4,0 [−4,0–−2,0]	0,147^a^
*Erkrankungsschwere*			
TISS 28	18,0 [14,0–24,0]	18,0 [14,5–21,5]	0,883^a^
SAPS II	36,0 [28,0–46,0]	38,0 [30,5–41,5]	0,937^b^
*BZ und Insulinbedarf*			
Glucose (mg/dl)	137,5 [121,5–153,3]	132,5 [117,0–168,0]	0,904^a^
Insulin (IE/h)	1,5 [0,0–5,0]	2,0 [0,0–4,0]	0,582^a^

*BPS* Behavioral Pains Scale, *TISS 28* Therapeutic Intervention Scoring System, *SAPS II* Simplified Acute Physiology Score II

^a^Mann-Whitney-Rangsummentest

^b^t‑Test. Ergebnisse sind als Median und 95 %-Konfidenzintervall angegeben

## Ergebnisse der logistischen Regression – Assoziation zwischen NFRT und Delir

Im logistischen Regressionsmodell wurde trotz geringer Fallzahl versucht, sich einem Schwellenwert zu nähern, ab dem eine Zu- oder Abnahme des Auftretens eines Delirs möglicherweise eintreten könnte. Nach Adjustierung für die entsprechenden Risikofaktoren wurde eine Abnahme des Auftretens eines Delirs bei einer NFRT < 20 mA gegenüber einem Reizschwellenkorridor von 20–40 mA wahrscheinlicher (OR 0,40, KI: 0,18–0,92, *p* = 0,03). Dieser Effekt blieb nach Adjustierung für potenzielle Störvariablen bestehen (Zusatzmaterial online: Tab. 4). Wurde der Reizschwellenkorridor, bei gleichbleibender unterer Schwelle, von < 20 mA auf Werte > 40 bzw. > 50 mA (20–50 mA und 20–60 mA) adjustiert, konnte eine tendenziell aufgetretene Reduktion der Delirinzidenz, wie oben beschrieben, nicht mehr beobachtet werden (Zusatzmaterial online: Tab. 4).

## Diskussion

Die Ergebnisse der vorliegenden, hypothesengenerierenden Studie geben erste Hinweise darauf, dass die Höhe der NFRT in einem Bereich über 50 mA möglicherweise mit einer Mortalitätszunahme assoziiert sein könnte. Die Delirinzidenz scheint bei niedrigen NFRT-Werten im Bereich um 20 mA beim Intensivpatienten u. U. abzunehmen.

### Assoziation der Höhe der NFRT als Surrogatparameter der Nozizeption mit der Mortalität und dem Auftreten eines Delirs

Mit steigender Dosis an Analgetika und Zunahme der Sedierungstiefe werden das Auftreten eines Delirs sowie eine gesteigerte Mortalität wahrscheinlicher [5, 31, 32].

Die verstorbenen Patienten der vorliegenden Studie haben signifikant mehr Opioide erhalten als das Kollektiv der nichtverstorbenen Patienten. Die statistisch ermittelte Reizschwelle > 50 mA, ab der eine Mortalitätssteigerung im untersuchten Kollektiv wahrscheinlicher wurde, ist möglicherweise auf die positive Assoziation der NFRT mit der Analgetikadosis zurückzuführen [8, 9, 14, 15, 34]. Die Messung des Schmerzniveaus erfolgte in der vorliegenden Studie mittels BPS, als standardisiertem Schmerzerfassungsinstrument, ergänzt um die NFRT-Messung [19]. In einer Analyse des prospektiven Datensatzes hat sich gezeigt, dass die BPS mit der Höhe der NFRT negativ assoziiert ist, was sich, wenngleich ohne statistisch Signifikanz, aus der vorliegenden retrospektiven Analyse ebenfalls ableiten lässt [23]. In einem heterogenen Kollektiv kritisch kranker Patienten müssen aber zwingend weitere Faktoren, die die Höhe der NFRT beeinflussen können, berücksichtigt werden. So ist anzunehmen, dass die beim Intensivpatienten häufig auftretende Polyneuro- und Myopathie die NFRT durch eine Störung der Nervenleitgeschwindigkeit bzw. der muskulären Reizantwort potenziell beeinflussen kann [6, 17, 28]. Als ein möglicher Risikofaktor für die Entwicklung einer Critical-Illness-Polyneuro- und Myopathie (CIP, CIM) gilt dabei die Hyperglykämie [24]. Die Gruppe der verstorbenen Patienten hatte statistisch signifikant höhere Blutzuckerkonzentrationen und entsprechend höhere kumulative Insulindosen, die das Vorliegen einer CIP-CIM begünstigen können. Knapp zwei Drittel der untersuchten Patienten waren männlich. France et al. konnten eine geschlechterspezifische Abhängigkeit der NFRT nachweisen. Frauen wiesen dabei u. a. niedrigere Reizschwellen zur Auslösung des NFR auf als Männer [13]. Verschiedene Studien haben den Einfluss des Geschlechtes auf das Outcome anästhesiologisch/intensivmedizinischer Patienten untersucht, mit teils gegensätzlichen Ergebnissen [17, 33]. Ob ein Zusammenhang zwischen dem Geschlecht, der Höhe der NFRT und dem Outcome oder der Häufigkeit des Auftretens eines Delirs besteht, kann anhand der vorliegenden Daten nicht beantwortet werden.

Neben der Mortalität wurde die Assoziation der NFRT mit dem Auftreten eines Delirs untersucht. Bei niedrigeren Reizschwellen, also weniger Analgetika und/oder Sedativa, wurde eine Tendenz zu einer Reduktion des Auftretens eines Delirs beobachtet. Wie in verschiedenen Studien berichtet, führt eine Reduktion der Sedierungstiefe zu einer verminderten Delirinzidenz bei Intensivpatienten [27, 28]. Schwieriger ist die Anpassung der Analgetika bei nichtmitteilungsfähigen Patienten. Eine Anpassung der Analgetikadosis auf niedrige NFRT-Werte würde, da kein Schwellenwert existiert, ab dem Schmerzfreiheit angenommen werden kann, potenziell schmerzhafte Zustände in Kauf nehmen. Schmerz und Nozizeption führen zu einer exzessiven Stressreaktion, die über verschiedene Mechanismen u. a. metabolische und neuroendokrine Vorgänge negativ beeinflussen kann [12]. Beispielsweise steigt das Risiko für Wundinfektionen und für die Entwicklung chronischer Schmerzzustände [20].

Das Alter der Patienten beeinflusst das Auftreten eines Delirs, ist aber, aufgrund entsprechend häufiger Komorbiditäten, häufig auch mit einer höheren Mortalität im Rahmen einer kritischen Erkrankung assoziiert [7]. Das Alter beeinflusst aber auch die Höhe der NFRT. Sandrine et al. verglichen beispielsweise die Höhe der NFRT bei Schulkindern mit derjenigen junger Erwachsener. Je jünger die Probanden waren, desto geringer war die Höhe der NFRT, was mit Unterschieden in der inhibitorischen Schmerzleitung von Jugendlichen gegenüber Erwachsenen erklärt wurde [23]. Ob beim kritisch kranken Patienten unter Berücksichtigung der oben genannten Einflussfaktoren das Alter Auswirkungen auf die NFRT haben kann, wurde bislang nicht untersucht.

### Limitationen

Schlussendlich ist die Messung der NFRT in einem heterogenen Patientenkollektiv kritisch kranker Patienten vielen verschiedenen, potenziellen Störfaktoren unterworfen, die eine alleinige Reduktion des Analgosedierungsregimes nur anhand der Höhe der NFRT zum aktuellen Zeitpunkt nicht erlauben. Die oben diskutierten Einflussfaktoren auf die NFRT wie beispielsweise eine potenzielle CIP-CIM, das Patientenalter und -geschlecht müssen in der Interpretation der Ergebnisse berücksichtigt werden. Das retrospektive Studiendesign sowie die kritische Erkrankung der Patienten machte es unmöglich, eine Ausgangsmessung der individuellen Reizschwelle vor Eintreten der jeweiligen Erkrankung der Patienten zu erheben. Die Heterogenität des Patientenkollektives führte dazu, dass nicht in jedem Reizschwellenkorridor für jeden Einflussfaktor adjustiert werden konnte. Durch die geringe Fallzahl der aufgrund der SARS-CoV-2-Pandemie vorzeitig abgebrochenen Studie konnten keine Subgruppenanalysen an Patienten mit vergleichbaren Krankheitsbildern etc. durchgeführt werden, was eine bessere Interpretation der Ergebnisse im heterogenen Patientenkollektiv erlaubt hätte.

## Schlussfolgerung

Die Messung des NFRT in einem heterogenen Kollektiv kritisch kranker Patienten zeigt eine Tendenz zu einer Mortalitätssteigerung bei höheren Werten sowie eine Tendenz zu einer Reduktion der Delirinzidenz bei niedrigeren Werten. Da die Messung der NFRT vielen verschiedenen Einflussfaktoren unterliegt, die im Rahmen einer intensivmedizinischen Komplexbehandlung nicht standardisiert erfasst und kontrolliert werden können, ist eine Anpassung des Analgosedierungsregimes, basierend auf der gemessenen NFRT, zum aktuellen Zeitpunkt obsolet. Ob und wie die Höhe der NFRT mit der Mortalität und Delirinzidenz korreliert, muss in kontrollierten Studien an kritisch kranken Patienten weiter untersucht werden.

## Fazit für die Praxis

- Nicht nur die zu tiefe Sedierung, sondern auch eine zu exzessive Analgesie hat negative, Outcome-relevante Auswirkungen auf den kritisch kranken Patienten.
- Die NFRT-Messung beim kritisch kranken Patienten unterliegt vielen verschiedenen Einflussfaktoren, wie u. a. einer möglichen CIP-CIM; diese können die Ergebnisse beeinflussen.
- Eine Anpassung der Analgosedierung anhand der Höhe der NFRT kann aktuell nicht empfohlen werden.

## Supplementary Information




