# Supplementary material for: Potential effect of the stimulus threshold level of the nociceptive flexion reflex (NFRT) on mortality and delirium incidence in the critically ill patient: a retrospective cohort analysis
Source: Anaesthesiologie. 2022 Sep 27;71(12):921–9. [Article in German] doi: 10.1007/s00101-022-01206-8 (PMC9514181; doi:10.1007/s00101-022-01206-8)
Supplement: Supplementary file 1 [file 101_2022_1206_MOESM1_ESM.pdf]

Zusatzmaterial zum Beitrag „Potentieller Einfluss der Reizschwellenhöhe des nozizeptiven Flexionsreflexes auf die Mortalitäts- und Delirinzidenz beim kritisch kranken Patienten: eine retrospektive Kohortenanalyse" von Schick B, Schmid S, Mayer B et al. (2022) in *Die Anaesthesiologie*
Beitrag und Zusatzmaterial stehen Ihnen auf www.springermedizin.de zur Verfügung. Bitte geben Sie dort den Beitragstitel in die Suche ein.

- Tabelle 1 - Übersicht über die Anzahl der Patienten und die Häufigkeit der NFRT-Messungen pro Reizschwellenkorridor.
- Tabelle 2 – Vergleich der Höhe der NFRT nicht verstorbener und verstorbener, sowie nicht deliranter und deliranter Patienten.
- Tabelle 3 - Übersicht der Odds Ratios - NFRT und Mortalität, adjustiert für die aufgeführten Einflussfaktoren, getrennt nach Vergleich der Reizschwellenkorridore der NFRT.
- Tabelle 4 - Übersicht der Odds Ratios - NFRT und Delir, adjustiert für die aufgeführten Einflussfaktoren, getrennt nach Vergleich der Reizschwellenkorridore der NFRT.

Tabelle 1 - Übersicht über die Anzahl der Patienten und die Häufigkeit der NFRT-Messungen pro Reizschwellenkorridor.

Reizschwellenkorridor	Anzahl an NFRT-Messungen	Gesamtanzahl an Patienten je NFRT-Korridor	Mittlere Anzahl an NFRT-Messungen je Patient
**Alle Patienten (n = 57)**			
**< 20,0 mA**	95,0	35,0	2,7
**20,0–40,0 mA**	93,0	30,0	3,1
**> 40,0 mA**	109,0	34,0	3,2
**20,0–50,0 mA**	125,0	36,0	3,5
**> 50,0 mA**	77,0	27,0	2,9
**20,0–60,0 mA**	141,0	36,0	4,0
**> 60,0 mA**	61,0	22,0	2,8

NFRT: Nociceptive flexion reflex threshold/nozizeptive Flexionsreflexschwelle
Ergänzend ist die Anzahl an Patienten angegeben, bei denen die jeweilige NFRT mindestens einmal gemessen werden konnte sowie die durchschnittliche Anzahl an Messungen, je Patient.

Tabelle 2 – Vergleich der Höhe der NFRT nicht verstorbener und verstorbener, sowie nicht deliranter und deliranter Patienten.

	NFRT (mA) Median/Mittelwert	Konfidenzintervall/Standardabweichung	p-Wert
**Nicht verstorben versus verstorben**			
Tag 1 versus<br>Tag 1$_{†}$	69,00<br>51,60	22,00–91,00<br>25,65–89,50	0,765[1]
Tag 2 versus<br>Tag 2$_{†}$	51,60<br>31,50	25,65–89,50<br>25,73–39,08	0,264[1]
Tag 3 versus Tag3$_{†}$	82,00<br>39,00	36,68–90,00<br>32,00–59,60	0,139[1]
Tag 4 versus Tag<br>4$_{†}$	46,13<br>62,30	42,02<br>35,38	0,560[2]
**Nicht delirant versus delirant**			
Tag 1 versus<br>Tag 1$_{Delir}$	36,90<br>40,00	25,00–50,60<br>22,38–84,30	0,587[1]
Tag 2 versus<br>Tag 2$_{Delir}$	28,57<br>34,70	25,18–35,92<br>26,50–88,00	0,081[1]
Tag 3 versus<br>Tag 3$_{Delir}$	39,00<br>69,00	32,00–58,60<br>27,65–95,50	0,184[1]
Tag 4 versus<br>Tag 4$_{Dellir}$	66,47<br>49,26	48,85<br>24,98	0,510[2]

†: verstorben, [1]Mann-Whitney-U-Test, [2]gepaarter t-Test, NFRT = Nociceptive flexion reflex threshold/nozizeptive Flexionsreflexschwelle.

Tabelle 3 - Übersicht der Odds Ratios - NFRT und Mortalität, adjustiert für die aufgeführten Einflussfaktoren, getrennt nach Vergleich der Reizschwellenkorridore der NFRT.

NFRT adjustiert für	OR > 40 vs. 20–40 mA	Konfidenzintervall		P
Mortalität	1,9277	0,5779	6,4306	0,2856
Alter	1,9067	0,5752	6,3200	0,2912
Geschlecht	1,7807	0,5193	6,1067	0,3588
Größe	2,0109	0,5876	6,8816	0,2658
TISS-28	1,0003	0,1712	5,8442	0,9998
SAPSII	1,2452	0,3405	4,5534	0,7403
RASS	1,3389	0,3907	4,5880	0,6423
BPS	1,9088	0,5564	6,5480	0,3040
Remifentanil	0,7788	0,2297	2,6408	0,6882
**NFRT adjustiert für**	**OR > 50 vs. 20–50 mA**	**Konfidenzintervall**		**P**
Alter	3,3248	0,8952	12,3481	0,0727
Geschlecht	2,7942	0,7158	10,9074	0,1392
Größe	3,0372	0,8001	11,5295	0,1026
TISS-28	2,0615	0,3615	11,7572	0,4154
SAPSII	2,386	0,6857	12,5933	0,1466
RASS	2,2168	0,5957	8,2489	0,2351
BPS	3,2967	0,8587	12,6558	0,0822
Remifentanil	1,4667	0,3986	5,3974	0,5645
**NFRT adjustiert für**	**OR > 60 vs. 20–60 mA**	**Konfidenzintervall**		**P**
Mortalität	3,5875	0,9067	14,1946	0,0687
Alter	3,5654	0,9054	14,0409	0,0691
Geschlecht	3,0721	0,7396	12,7608	0,1224
Größe	3,3463	0,8169	13,7078	0,0932
TISS-28	2,6835	0,4744	15,1784	0,2642
SAPSII	4,1441	0,9692	17,7198	0,0551
RASS	2,5856	0,6666	10,0287	0,1696
BPS	3,5676	0,8912	14,2824	0,0723
Remifentanil	1,6607	0,4013	6,8727	0,4839
**NFRT adjustiert für**	**OR < 20 vs. 20–40 mA**	**Konfidenzintervall**		**P**
Mortalität	1,2463	0,6425	2,4177	0,5148
Alter	1,2713	0,6824	2,3684	0,4496
Geschlecht	1,3426	0,6849	2,6320	0,3910
Größe	1,5368	0,7690	3,0714	0,2238
TISS-28	0,3689	0,2221	1,9732	0,4592
SAPSII	0,8579	0,3226	2,2812	0,7587
RASS	1,5654	0,8237	2,9750	0,1713
BPS	1,2667	0,6419	2,4994	0,4954
Remifentanil	0,4931	0,1732	1,4040	0,1854
**NFRT adjustiert für**	**OR < 20 vs. 20–50 mA**	**Konfidenzintervall**		**P**
Mortalität	1,4852	0,7180	3,0725	0,2862
Alter	1,5260	0,7743	3,0077	0,2221
Geschlecht	1,5574	0,7625	3,1809	0,2241
Größe	1,7091	0,7948	3,6751	0,1700
TISS-28	0,9491	0,3184	2,8292	0,9253
SAPSII	1,2161	0,4673	3,1646	0,6885
RASS	1,8576	0,9137	3,7768	0,0871
BPS	1,5075	0,7098	3,2017	0,2855
Remifentanil	0,6382	0,2089	1,9503	0,4307
**NFRT adjustiert für**	**OR < 20 vs. 20–60 mA**	**Konfidenzintervall**		**P**
Mortalität	1,384	0,6252	3,0637	0,4229
Alter	1,4204	0,6702	3,0107	0,3598

Geschlecht	1,4806	0,6696	3,274	0,3324
Größe	1,6149	0,6969	3,7423	0,2637
TISS-28	1,0429	0,3558	3,0563	0,939
SAPSII	1,3218	0,5272	3,3141	0,5519
RASS	1,8539	0,8472	4,0569	0,1224
BPS	1,1411	0,6138	3,2455	0,4173
Remifentanil	0,6354	0,2078	1,9428	0,4264

OR: Odds ratio, P: p-Wert, TISS-28: Therapeutic Intervention Scoring System-28, SAPSII: Simplified Acute Physiology Score II, RASS: Richmond Agitation Sedation Scale, BPS: Behavioral Pain Scale.

Tabelle 4 - Übersicht der Odds Ratios - NFRT und Delir, adjustiert für die aufgeführten Einflussfaktoren, getrennt nach Vergleich der Reizschwellenkorridore der NFRT.

NFRT adjustiert für	OR > 60 vs. 20–60 mA	Konfidenzintervall		P
Delir	0,6596	0,1687	2,5793	0,5497
Alter	0,6093	0,1418	2,6179	0,5054
Geschlecht	0,4546	0,1174	1,7606	0,2538
Größe	0,5695	0,1587	2,0438	0,3878
TISS-28	0,2995	0,0789	1,1372	0,0756
SAPSII	0,2621	0,0657	1,0458	0,0579
RASS	0,7352	0,1794	3,013	0,6691
BPS	0,6681	0,1693	2,6371	0,5648
Sufentanil	0,5718	0,0765	4,2723	0,5859
Remifentanil	0,488	0,0747	3,1869	0,4537
**NFRT adjustiert für**	**OR > 50 vs. 20–50 mA**	**Konfidenzintervall**		**P**
Delir	0,7615	0,2590	2,2385	0,6204
Alter	0,7161	0,2195	2,3361	0,5799
Geschlecht	0,5048	0,1756	1,4513	0,2046
Größe	0,6473	0,2387	1,7549	0,3927
TISS-28	0,5706	0,1558	2,0902	0,3970
SAPSII	0,4813	0,1397	1,6583	0,2466
RASS	0,8767	0,2971	2,5874	0,8117
BPS	0,7765	0,2610	2,3106	0,6493
Sufentanil	0,3813	0,0685	2,1219	0,2709
Remifentanil	0,8585	0,2171	3,3955	0,8278
**NFRT adjustiert für**	**OR > 40 vs. 20–40 mA**	**Konfidenzintervall**		**P**
Delir	0,6687	0,3158	1,4159	0,2931
Alter	0,6086	0,2609	1,4196	0,2505
Geschlecht	0,5258	0,2244	1,2318	0,1389
Größe	0,6592	0,3127	1,3896	0,2734
TISS-28	0,5864	0,2344	1,4667	0,2538
SAPSII	0,4994	0,2162	1,1538	0,1041
RASS	0,7382	0,3456	1,5768	0,4331
BPS	0,6826	0,3133	1,4873	0,3366
Sufentanil	0,4133	0,1000	1,7082	0,2223
Remifentanil	0,7405	0,2619	2,0939	0,5711
**NFRT adjustiert für**	**OR < 20 vs. 20–60 mA**	**Konfidenzintervall**		**P**
Delir	0,4406	0,1648	1,178	0,1024
Alter	0,4255	0,1759	1,0293	0,058
Geschlecht	0,4311	0,1746	1,0645	0,0681
Größe	0,4797	0,1828	1,2588	0,1356
TISS-28	0,2613	0,0744	0,9171	0,0362
SAPSII	0,2776	0,0751	1,0258	0,0546
RASS	0,4051	0,1405	1,1682	0,0945
BPS	0,4204	0,1467	1,2047	0,1067
Sufentanil	0,9475	0,3387	2,6501	0,9181
Remifentanil	0,639	0,1591	2,566	0,5278
**NFRT adjustiert für**	**OR < 20 vs. 20–50 mA**	**Konfidenzintervall**		**P**
Delir	0,4495	0,1724	1,1719	0,1019
Alter	0,4362	0,1879	1,0130	0,0536
Geschlecht	0,4201	0,1777	0,9930	0,0482
Größe	0,4805	0,1884	1,2259	0,1251
TISS-28	0,3007	0,0842	1,0739	0,0643
SAPSII	0,3042	0,0824	1,1233	0,0742

RASS	0,4176	0,1482	1,1770	0,0986
BPS	0,4305	0,1561	1,1878	0,1036
Sufentanil	0,7859	0,2764	2,2347	0,6514
Remifentanil	0,7097	0,1865	2,7006	0,6151
**NFRT adjustiert für**	**OR < 20 vs. 20–40 mA**	**Konfidenzintervall**		**P**
Alter	0,3790	0,1816	0,7913	0,0098
Geschlecht	0,3908	0,1801	0,8464	0,0172
Größe	0,4520	0,2005	1,0191	0,0556
TISS-28	0,2793	0,0856	0,9115	0,0345
SAPSII	0,2782	0,0821	0,9426	0,0399
RASS	0,3782	0,1517	0,9428	0,0369
BPS	0,387	0,1601	0,9356	0,0350
Sufentanil	0,6992	0,2281	2,1429	0,5312

OR: Odds ratio, P: p-Wert, TISS-28: Therapeutic Intervention Scoring System-28, SAPSII: Simplified Acute Physiology Score II, RASS: Richmond Agitation Sedation Scale, BPS: Behavioral Pain Scale.
